# P-212. The Effects of Magnesium Sulfate on Mortality and Morbidity of Adult Patients with Tetanus in San Lazaro Hospital, Manila, Philippines from January 2012 to December 2021: A Retrospective Cohort Study

**DOI:** 10.1093/ofid/ofaf695.434

**Published:** 2026-01-11

**Authors:** Greco Mark B Malijan, Ana Ria L Sayo

**Affiliations:** University of Oxford, London, England, United Kingdom; San Lazaro Hospital, Manila, National Capital Region, Philippines

## Abstract

**Background:**

Tetanus causes significant morbidity and mortality worldwide, despite effective vaccines. It results from tetanus toxin (TeNT), leading to muscle rigidity, spasms, and autonomic instability. Endemic in the Philippines, it remains a major health challenge. This study assesses the impact of magnesium sulfate on mortality, ventilator use, and hospital stay in tetanus patients.

**Figure:**
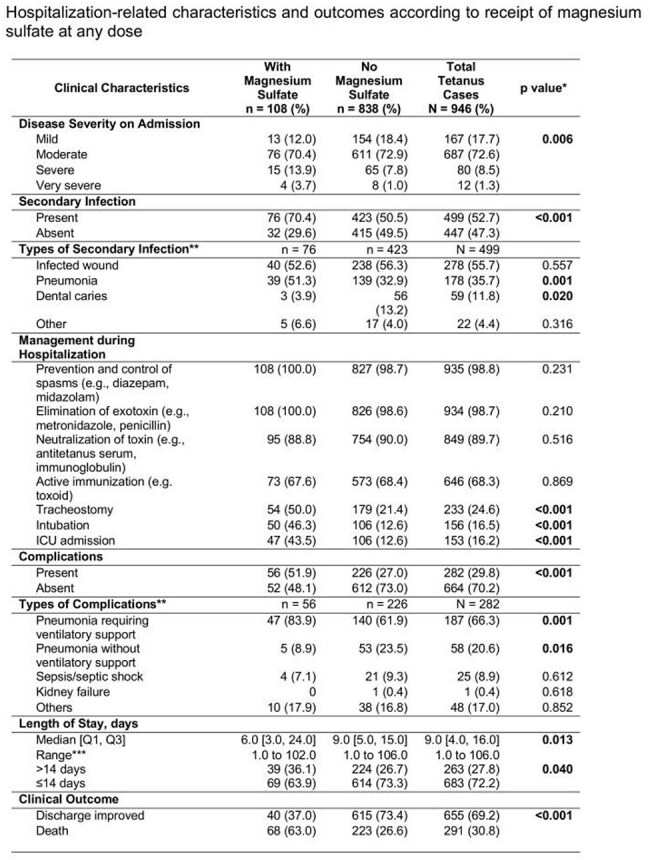

**Figure:**
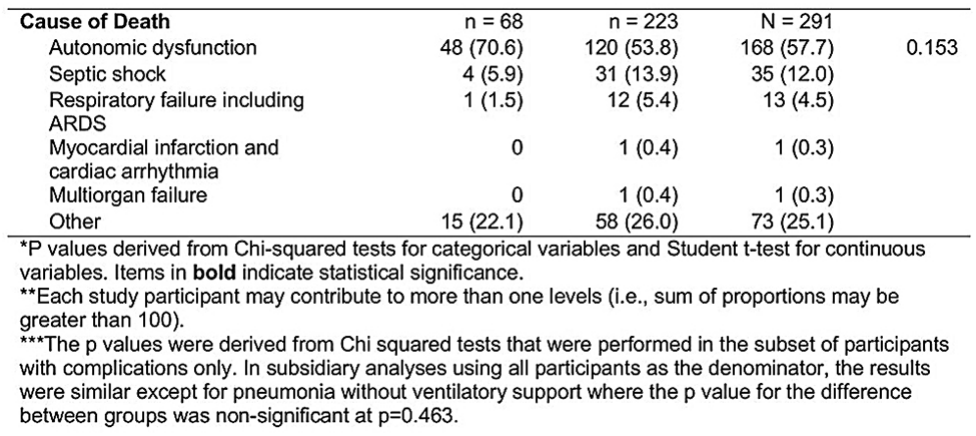

**Methods:**

Retrospective cohort study which focused on hospitalized adult tetanus patients (2012-2021) in an Infectious Diseases referral hospital in Manila, the Philippines. It involved review of patients chart examining clinical features, and the effects of magnesium sulfate on treatment outcomes.

**Figure:**
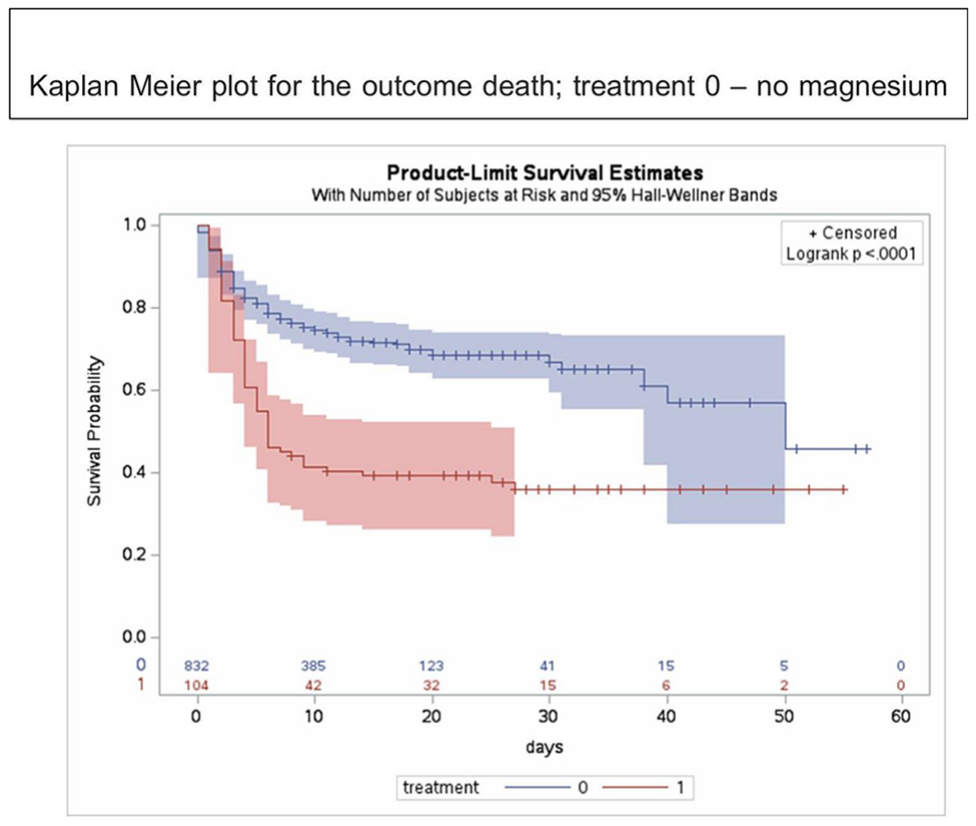

**Results:**

A total of 946 patients, mostly middle-aged males and laborers from urban and nearby regions, were included. Most presented with moderate disease severity. Patients receiving magnesium sulfate had higher rates of severe and very severe tetanus and required more respiratory interventions, such as tracheostomy, intubation, and ICU admission. The median hospital stay was 9 days overall; magnesium recipients had a shorter median stay, though prolonged hospitalization was more common among them. The overall mortality rate was 30.8%, with a significantly higher death rate in the magnesium group (63% vs. 26.6%, p< 0.001). Autonomic dysfunction was the leading cause of death (57.7%) and was more common among magnesium recipients, though not statistically significant. Cox regression showed magnesium sulfate was associated with increased need for mechanical ventilation (HR 2.58, 95% CI 1.43–4.27), but no significant association was found with mortality or longer hospital stay.

**Conclusion:**

The high case fatality rate persists despite holistic care, with autonomic dysfunction as the main cause of death in both groups. Developing standardized treatment protocols, training healthcare workers for early recognition, prompt intervention, and conducting further research are essential. Public Health should focus on strengthening catch-up vaccination. Improving access to timely care through local facilities or transfers system are crucial.

**Disclosures:**

All Authors: No reported disclosures

